# Syphilitic descending aortic aneurysm presenting with characteristic double-ring sign on CT: Radiologic–pathologic correlation

**DOI:** 10.1016/j.radcr.2026.06.092

**Published:** 2026-07-07

**Authors:** Tsubasa Sugie, Syohei Miyaguni, Nanae Tsuchiya, Yuma Chinen, Hitoshi Inafuku, Tatsuya Maeda, Kojiro Furukawa, Naoki Wada, Akihiro Nishie

**Affiliations:** a Department of Radiology, Graduate School of Medicine, University of the Ryukyus, Okinawa, Japan; b Department of Thoracic and Cardiovascular Surgery, Graduate School of Medicine, University of the Ryukyus, Okinawa, Japan; c Department of Pathology and Oncology, Graduate School of Medicine, University of the Ryukyus, Okinawa, Japan

**Keywords:** Syphilitic aortitis, Syphilitic aortic aneurysm, Computed tomography, Double-ring sign, Radiologic–pathologic correlation

## Abstract

A man in his 50s presented with worsening anterior chest pain. Serologic testing revealed active syphilis infection, and polymerase chain reaction testing was positive for COVID-19. Contrast-enhanced computed tomography demonstrated a descending thoracic aortic aneurysm with circumferential wall thickening and a characteristic double-ring sign suggestive of inflammatory aortitis. After blood pressure control and antibiotic therapy, elective descending aortic replacement was performed. Surgical inspection showed diffuse intimal thickening with a tree-bark appearance. Histopathology demonstrated lymphoplasmacytic infiltration in the media, adventitia, and around the vasa vasorum, consistent with syphilitic aortitis. This case highlights the diagnostic value of CT, particularly the double-ring sign, in identifying syphilitic aortitis and demonstrates a rare presentation of syphilitic aneurysm in the descending thoracic aorta.

## Introduction

Syphilitic aortitis represents a late manifestation of untreated *Treponema pallidum* infection and remains an important cause of thoracic aortic aneurysm despite the antibiotic era [[1], [2], [3]]. Cardiovascular involvement typically develops decades after primary infection due to progressive inflammation of the vasa vasorum [1]. Following syphilitic infection, *Treponema pallidum* spreads through the lymphatic tissue surrounding the vasa vasorum from the adventitia into the aortic media [1]. The resulting medial necrosis weakens the aortic wall, leading to aneurysm formation. Syphilitic aortic aneurysms are commonly saccular and are characterized by mural thrombi and a classic “tree-bark” appearance of the intimal surface [4].

CT plays a central role in the evaluation of aortic aneurysms and allows assessment of both luminal morphology and aortic wall abnormalities [5]. Syphilitic aneurysms most commonly involve the ascending aorta (approximately 50%), followed by the aortic arch (30-40%), whereas involvement of the descending thoracic aorta is relatively uncommon (10-15%) and abdominal aortic involvement is rare (< 5%) [2]. CT findings of syphilitic aortitis include aneurysmal dilatation of the ascending thoracic aorta, circumferential aortic wall thickening, and periaortic inflammatory changes. Aortic inflammation may manifest as concentric mural thickening with a layered appearance resembling the “double-ring sign,” reflecting involvement of the aortic media and adventitia. Associated findings include coronary ostial stenosis, aortic regurgitation, and saccular aneurysm formation with mural thrombus [[5], [6], [7]].

We report a rare case of syphilitic aneurysm arising in the descending thoracic aorta, demonstrating a characteristic double-ring sign on CT and excellent radiologic–pathologic correlation.

## Case presentation

### History of present illness

A man in his 50s presented to the emergency department with worsening anterior chest pain. He had intermittently experienced anterior chest pain for approximately 4-5 years. Ten days before presentation, the pain became persistent, and on the day of admission, the pain acutely worsened, prompting emergency department evaluation. The pain was described as a pressing sensation and became more severe with physical activity. There was no radiation of pain or associated dyspnea. He denied fever, sore throat, cough, or rhinorrhea.

### Past medical history

The patient had a history of hypertension and hyperuricemia identified during routine health checkups. Although antihypertensive therapy had been initiated previously, he had discontinued follow-up and treatment. The patient did not recall any prior symptoms suggestive of early symptomatic syphilis, such as chancre, rash, fever, or malaise, and was unaware of any possible history of syphilis infection.

### Physical examination

Height: 180 cm; weight: 77 kg. Body temperature was 36.6°C. Pulse rate was 84 beats/min with regular sinus rhythm. Blood pressure was elevated at 189/108 mmHg. Respiratory rate was 21 breaths/min, and oxygen saturation was 98% on room air. Cardiac auscultation revealed regular heart sounds without murmurs. Pulmonary auscultation demonstrated no wheezes, crackles, or stridor. Bilateral radial pulses were symmetric and regular, with no pulse deficit.

### Laboratory tests

Serologic testing for syphilis was positive, with a rapid plasma reagin level of 130 R.U. and a *Treponema pallidum* latex agglutination level of 5800 T.U.. Reverse transcription polymerase chain reaction testing for COVID-19 was also positive. Tests for HIV, hepatitis B, and hepatitis C were negative. C-reactive protein (CRP) was mildly elevated at 3.54 mg/dL, serum IgG level was within the normal range, although serum IgG4 level was not assessed.

### Imaging findings

**Fig. 1:**
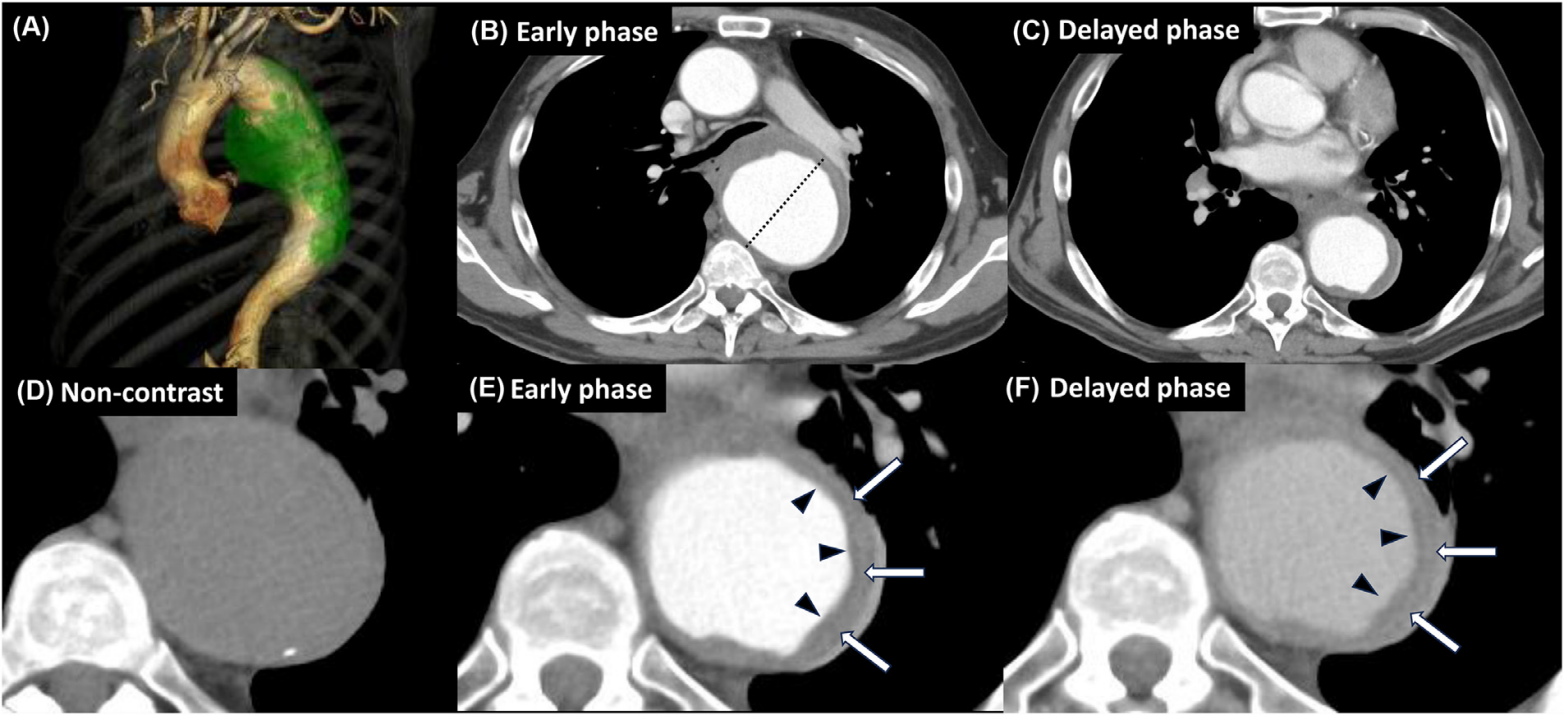
(A) Volume-rendered image. (B, C) Early-phase images. (D–F) Magnified views of (C), including non-contrast (D), early phase (E), and delayed phase (F). Contrast-enhanced CT demonstrates a descending thoracic aortic aneurysm with circumferential wall thickening. The intimal layer shows poor enhancement (arrow head), whereas the outer medial and adventitial layers demonstrate ring-like enhancement (arrow) (double-ring sign), suggestive of inflammatory aortitis. The aneurysm measured 70 mm in maximum diameter (dotted line).

**Fig. 2:**
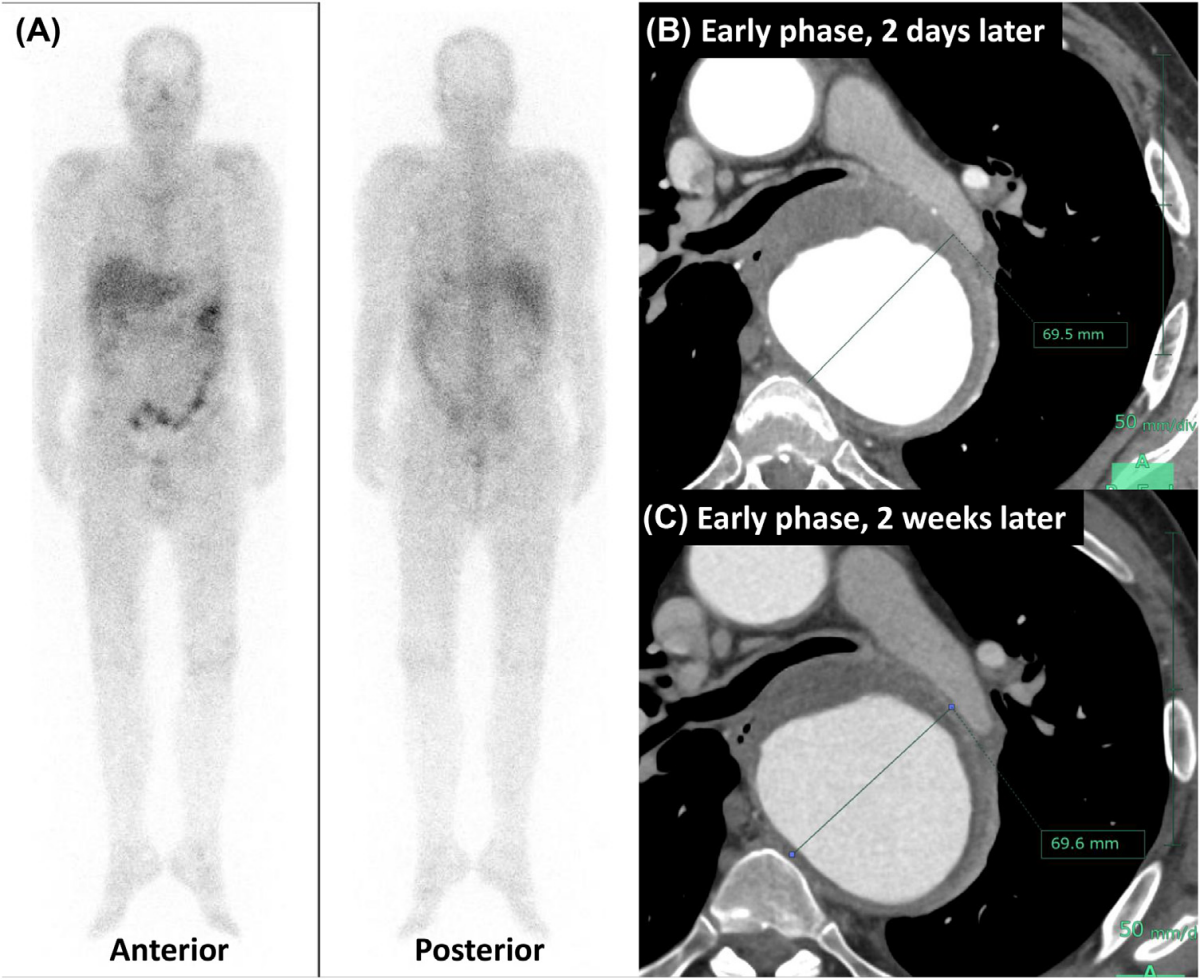
(A) Anterior and posterior whole-body gallium scintigraphy images. No abnormal uptake is observed in the descending thoracic aorta or elsewhere suggestive of active systemic inflammatory disease. (B, C) Follow-up contrast-enhanced CT early-phase images obtained 2 days (B) and 2 weeks (C) after the initial examination. The maximum aneurysm diameter remained unchanged (69.5 mm in [B] and 69.6 mm in [C]), indicating no significant interval enlargement.

Contrast-enhanced CT demonstrated a descending thoracic aortic aneurysm measuring 70 mm in diameter. The aortic wall showed circumferential thickening. The intimal layer exhibited poor enhancement, whereas the outer medial and adventitial layers demonstrated ring-like enhancement on delayed-phase imaging, producing a characteristic double-ring sign suggestive of inflammatory aortitis (Fig. 1). Gallium scintigraphy demonstrated no abnormal uptake in the descending thoracic aorta or other sites suggestive of active systemic inflammatory disease (Fig. 2A). Based on imaging findings and serologic results, syphilitic aortitis was strongly suspected. Chest CT showed no pulmonary findings suggestive of COVID-19 pneumonia.

### Clinical course and treatment

Antihypertensive therapy and intravenous penicillin treatment were initiated. Chest pain resolved rapidly under blood pressure control. Follow-up CT performed during hospitalization showed no significant interval change in aneurysm size (Fig. 2B-C). Surgery was deferred until resolution of active COVID-19 infection to reduce perioperative complications while strict blood pressure control was maintained because no imaging findings suggestive of impending rupture were observed, and gallium scintigraphy demonstrated no evidence of active aortic inflammation. After confirmation of COVID-19 resolution, elective descending thoracic aortic replacement was performed on hospital day 29.

### Surgical findings

**Fig. 3:**
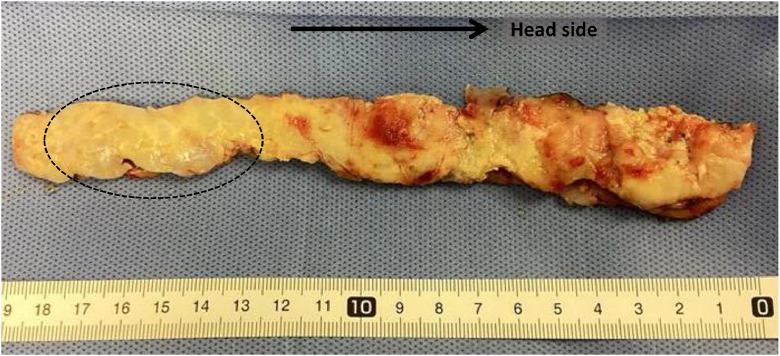
Resected aneurysmal aortic wall specimen demonstrating diffuse intimal thickening and characteristic wrinkling (circle) (“tree-bark appearance”), a classic gross pathological feature of syphilitic aortitis.

Intraoperative inspection revealed diffuse whitish intimal thickening without mural thrombus formation (Supplemental Video 1). The intimal surface demonstrated a characteristic wrinkled or “tree-bark” appearance, suggestive of syphilitic aortitis (Fig. 3).

### Pathological findings

**Fig. 4:**
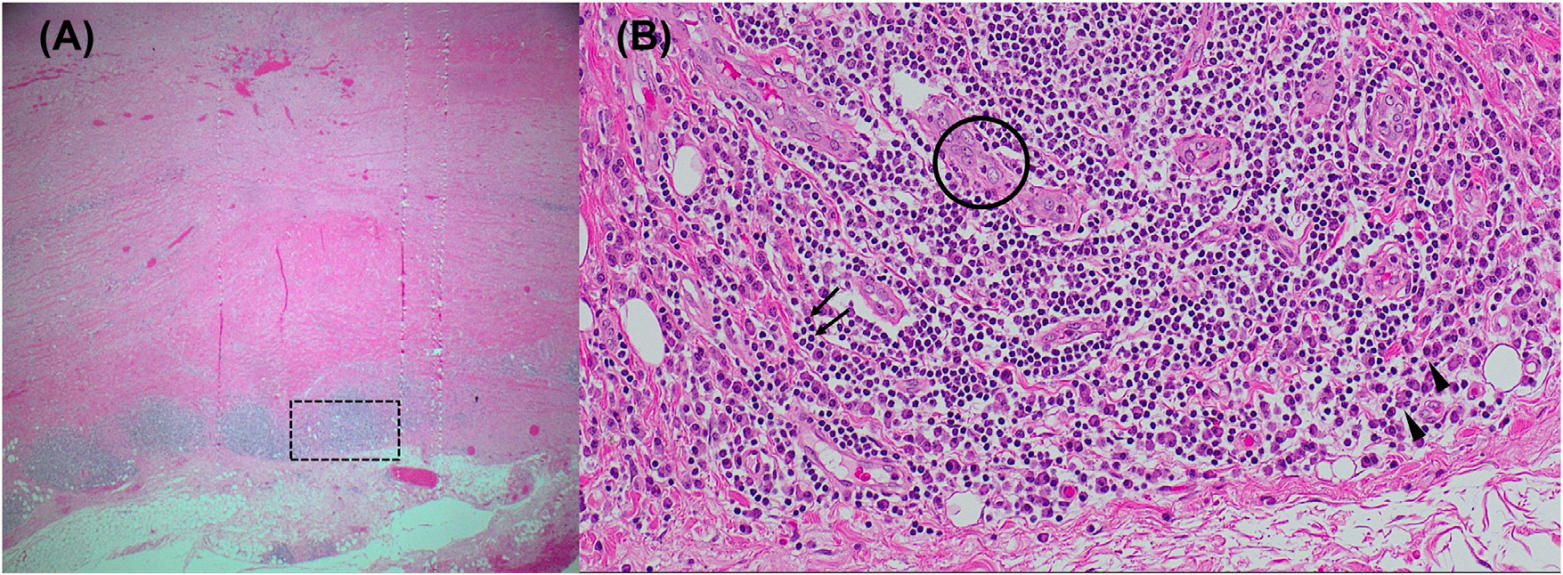
Histopathological examination shows lymphocyte and plasma cell–predominant inflammatory infiltration accompanied by endothelial swelling of the vessels within the inflammatory focus. Plasma cells (arrowheads), lymphocytes (arrows), and endothelial swelling (circle) are identified. Note: Hematoxylin-eosin stain; low (a)- and high (b) -power views). The dotted rectangle in (a) indicates the range of the high power view.

Note: Hematoxylin–eosin stain; low (A)- and high (B) -power views). The dotted rectangle in (A) indicates the range of the high power view.

Histological examination revealed infiltration of lymphocytes and plasma cells in the media, adventitia, and around the vasa vasorum. In addition, endothelial cell swelling and fibrosis were observed (Fig. 4). Although spirochetes were not identified, these findings were consistent with syphilitic aortitis.

## Discussion

The present case demonstrated a rare syphilitic aneurysm arising in the descending thoracic aorta with a characteristic double-ring sign on CT and corresponding histopathological evidence of syphilitic aortitis.

Syphilitic aortitis results from obliterative endarteritis of the vasa vasorum leading to medial ischemia and progressive weakening of the aortic wall [1,2]. Although syphilis was historically a common cause of thoracic aortic aneurysm, its incidence markedly decreased after the introduction of antibiotics. However, the recent resurgence of syphilis necessitates renewed awareness among radiologists [3,8].

Syphilitic aneurysms most commonly involve the ascending aorta (approximately 50%), followed by the aortic arch (30-40%), whereas involvement of the descending thoracic aorta is relatively uncommon (10-15%) and abdominal aortic involvement is rare (< 5%) [1,2]. This distribution is thought to reflect the greater density of the vasa vasorum in the ascending thoracic aorta. Therefore, the present case represents an uncommon anatomical manifestation of cardiovascular syphilis.

Contrast-enhanced CT demonstrated circumferential wall thickening with a characteristic double-ring sign. This imaging appearance reflects inflammatory involvement of the aortic wall, in which the inflamed outer media and adventitia enhance while the edematous inner layer remains relatively hypoenhancing. Similar findings have been described in other forms of inflammatory aortitis, including Takayasu arteritis and IgG4-related disease [5,6]. Therefore, imaging findings alone are insufficient for definitive diagnosis, and correlation with clinical history, serologic testing, and pathological evaluation remains essential. The differential diagnosis of inflammatory thoracic aortic aneurysm includes Takayasu arteritis, giant cell arteritis, IgG4-related disease, and infectious (mycotic) aneurysms [5].

Radiologic–pathologic correlation further supports the imaging interpretation [9]. Histopathology demonstrated lymphoplasmacytic infiltration in the media, adventitia, and around the vasa vasorum with endothelial swelling, corresponding to the enhancing outer aortic wall observed on CT. Although spirochetes were not identified histologically, direct demonstration of *Treponema pallidum* is often difficult in tertiary syphilis because organisms are typically sparse within tissue [4,10]. Therefore, the diagnosis is commonly established on the basis of characteristic pathological findings together with positive serologic testing. In the present case, positive syphilis serology, obliterative vasculopathy of the vasa vasorum, lymphoplasmacytic inflammation, and characteristic intraoperative findings collectively supported the diagnosis of syphilitic aortitis.

COVID-19 infection was present at the time of diagnosis. COVID-19 has been associated with endothelial injury and systemic inflammatory activation, which may theoretically influence vascular inflammation [[11], [12]]. However, the relationship between COVID-19 infection and the clinical manifestation of syphilitic aortitis remains speculative, and a causal association cannot be established from a single case.

Several limitations should be acknowledged. Serum IgG4 levels were not measured; therefore, IgG4-related aortitis could not be completely excluded serologically, although the pathological and serological findings strongly supported syphilitic aortitis. In addition, FDG PET/CT was not performed, which may have provided further assessment of inflammatory activity and extent of disease.

## Conclusion

This case highlights the diagnostic value of CT, particularly the double-ring sign, in identifying inflammatory aortitis and demonstrates excellent radiologic–pathologic correlation in a rare case of syphilitic aneurysm arising in the descending thoracic aorta.

## Patient consent

Written informed consent was obtained from the patient featured in this case report.

## Appendix Supplementary materials

Supplemental Video 1 Intraoperative findings reveal diffuse intimal thickening with a characteristic wrinkled (``tree-bark'') appearance without mural thrombus formation. Image, video 1 dummy alt text
